# Long-Term Effect of Surgery in Graves' Disease: 20 Years Experience in a Single Institution

**DOI:** 10.1155/2015/542641

**Published:** 2015-05-12

**Authors:** Tae-Yon Sung, Yu-mi Lee, Jong Ho Yoon, Ki-Wook Chung, Suck Joon Hong

**Affiliations:** Department of Surgery, Asan Medical Center, University of Ulsan College of Medicine, Seoul 138-736, Republic of Korea

## Abstract

The present study compared the long-term outcome of subtotal thyroidectomy (ST) to that of total thyroidectomy (TT) in Graves' disease (GD). Patients with GD requiring surgery were divided between two groups: ST and TT. Postoperative thyroid function (PoTF) changes, including hypothyroidism, euthyroidism, and hyperthyroidism, and surgical complications were analyzed 3 months and 2 years after surgery. During the study period, 350 GD patients underwent surgery, of whom 254 underwent ST and 96 underwent TT. In the ST group, the rates of hypothyroidism, euthyroidism, and hyperthyroidism were 92.5%, 6.7%, and 0.4%, respectively, after 3 months, and 86.1%, 8.6%, and 5.3%, respectively, after 2 years. No difference in the rate of surgical complication was observed between the ST and TT groups (*p* = 0.089). Most of the ST patients showed hypothyroidism after surgery, and euthyroidism was rare. The long-term outcome of ST included noticeable PoTF changes and recurrence of GD. These results suggest that TT should be considered as a treatment option in GD requiring surgery.

## 1. Introduction

The three main treatment modalities for Graves' disease (GD) are antithyroid drug therapy, radioactive iodine therapy, and thyroid surgery [1]. Antithyroid drug therapy and radioactive iodine therapy are selected more frequently than surgery in early-stage GD [2–4]; however, in GD patients with symptomatic compression or large goiters, suspicion of malignancy, low uptake of radioactive iodine (RAI), a pregnancy planned within 6 months, or severe ophthalmopathy, thyroidectomy is the preferred modality despite the risks associated with surgery [5–7].

Thyroid surgery options for GD include subtotal thyroidectomy (ST) and total thyroidectomy (TT). In the past, ST, which preserves a functional thyroid remnant, was the standard choice, and many surgeons still prefer it. ST was also preferred because of its lower incidence of surgical complications than TT [8]. TT has been performed since the early 1960s [9] in limited cases including pediatric GD, serious side effects from antithyroid drugs, and suspicion of thyroid malignancy [10].

The advantages of ST include the maintenance of thyroid function, that is, euthyroidism, and the expectation of fewer surgical complications such as hypoparathyroidism and recurrent laryngeal nerve injury [5]; however, lack of complication after ST is not guaranteed by all surgeons, and hypothyroidism or hyperthyroidism recurrence rates differ between surgical techniques [8]. By contrast, TT prevents recurrence with certainty [11]. Recent studies showed that the incidence of surgical complications was not significantly different between ST and TT [11–15]; hence, we compared long-term postoperative thyroid function (PoTF) between ST and TT.

## 2. Methods

Consecutive GD patients requiring surgery between December 1995 and December 2010 were included in the study. A retrospective review of the clinical data was performed, and the patients were divided into two groups: the ST and TT groups. Patients showing low compliance during follow-up, such as patients who missed appointments or changed institution after surgery, and patients for whom surgical data were not available or did not include the weight of the remnant thyroid after ST were excluded. The diagnosis of GD was made when clinically enlarged thyroid goiter was found with thyroid associated ophthalmopathy and the measurement of thyroid-stimulation hormone binding inhibitor immunoglobulin to distinguish the reason of hyperthyroidism from others such as toxic multinodular goiter. Thyroid scan uptake examination was performed when in need. Thyroidectomy was performed by a single experienced endocrine surgeon (SJH). The surgical procedure was processed in the same manner for ST and TT. The diagnosis of GD was mainly performed from medical department by the TFT examinations and was sent for surgery when in need.

Two ST procedures were used: bilateral ST in which remnant thyroid was left at both upper pole areas and ipsilateral total thyroidectomy with contralateral ST (Hartley-Dunhill procedure). In ST, the amount of remnant thyroid was estimated by measuring a portion of resected thyroid cut into similar size and shape. The weight of the recut thyroid specimen that mimics the remnant thyroid was measured and recorded during surgery after each ST. The measurement was made by the operating surgeon based on his experience, the degree of hyperthyroidism, and thyroid gross findings. TT was recommended and performed mostly in younger patients and in patients with severe Graves' ophthalmopathy; however, the gradual change in surgical method preference from ST to TT over time was represented. Thyroid function tests (TFT) were performed after surgery in all cases, including T3 (98–180 ng/dL), thyroid-stimulating hormone (TSH) (0.4–5.0 *μ*U/mL), free T4 (fT4) (0.8–1.9 ng/dL), thyroglobulin (1.0–23.3 ng/mL), and anti-thyroglobulin antibody (0.0–60.0 U/mL). In ST, PoTF was measured to assess the change in thyroid function, and in TT, it was measured to determine the dosage of thyroid hormone. The first PoTF was measured 3 months after surgery, the second 6 months after surgery, and the third after 1 year of follow-up. Thereafter, the PoTF was measured yearly. At each visit, the need for thyroid hormone administration was assessed based on the PoTF measurement and symptoms of abnormal thyroid function. The thyroid hormone drug (or antithyroid hormone drug) was administered at any point when hypothyroidism or hyperthyroidism was diagnosed. The definition of hypothyroidism, euthyroidism, and hyperthyroidism was as follows: hypothyroidism (TFT including TSH above and fT4 below the normal range), euthyroidism (TFT including TSH and fT4 within the normal range), and hyperthyroidism (TFT including TSH below and fT4 above the normal range). To further assess changes in PoTF in ST, hypothyroidism and hyperthyroidism were subdivided into subclinical and clinical. Subclinical hypothyroidism was defined as hypothyroidism in TFT without thyroid hormone therapy, and clinical hypothyroidism was defined as hypothyroidism in TFT with thyroid hormone therapy. Subclinical hyperthyroidism was defined as hyperthyroidism in TFT without antithyroid drug therapy, and clinical hyperthyroidism was defined as hyperthyroidism in TFT with antithyroid drug therapy. PoTF was assessed for more than 2 years in the out-patient department to determine the long-term effect of ST. Recurrence of GD after surgery was defined as postoperative clinical hyperthyroidism requiring treatment with antithyroid drug therapy or RAI therapy after 2 years of follow-up. Surgical complications were compared between ST and TT including postoperative bleeding, recurrent laryngeal nerve injury, hypoparathyroidism, and wound seroma.

Statistical analysis was performed using SPSS Statistics, version 21 (IBM). Variables were compared by chi-square test or Fisher's exact test. A *p* ≤ 0.05 was considered significant. Data are reported as number (percentage), unless otherwise stated.

## 3. Results

**Figure 1:**
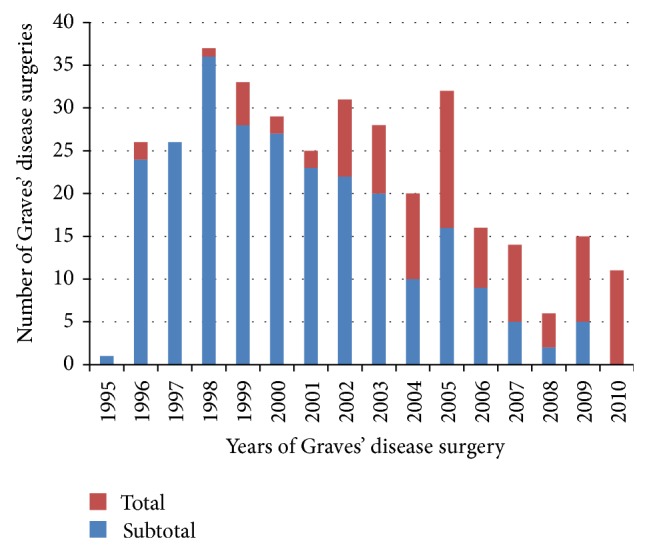
Changes in surgical method selected in Graves' disease requiring surgery.

Three hundred and sixty consecutive patients underwent thyroidectomy for GD, and 350 were enrolled in the study. Ten patients were excluded based on the criteria outlined in the methods. Of the 350 cases, 254 underwent ST and 96 underwent TT. In the TT group, six patients underwent initial ST and subsequent TT when final histopathology reported GD with occult thyroid carcinoma. The change in preferred surgical procedure from ST to TT that occurred over the years at the study institution shows a large increase in TT and a clear shift from ST to TT in 2010 (Figure 1).

The gender ratio showed a predominance of females over males (77.1 versus 22.9%). The median age at surgery was 30 years old. The median resected thyroid weight was 70.6 g, and the weight was larger in ST group than TT group, 84.9 g and 32.5 g, respectively, with statistical significance (*p* = 0.000). The median follow-up duration was 61.0 months. Ophthalmopathy was accompanied in 26.0% of the patients. The most frequent reasons for undergoing surgery for GD were resistance to antithyroid drug therapy (55.7%). The incidence of thyroid carcinoma presented in GD was 79 out of 350 patients (22.6%) of which 74 underwent TT. Of them, all presented as papillary thyroid carcinoma. The median size of papillary thyroid carcinoma was 0.83 cm (Table 1).

The PoTF 3 months after ST showed hypothyroidism in 235 patients (92.5%), euthyroidism in 17 patients (6.7%), and hyperthyroidism in 1 patient (0.4%). The ST group was divided into three subgroups based on remnant thyroid weight: <4.0 g (73 patients; 28.7%), ≥4.0 g and <6.0 g (153 patients; 60.2%), and ≥6.0 g (28 patients; 11.1%). A close relationship was observed between the weight of the thyroid remnant and PoTF: hypothyroidism was associated with smaller remnant thyroid, and hyperthyroidism was associated with larger remnant thyroid; however, the difference was not significant (*p* = 0.671) (Table 2). All TT patients showed hypothyroidism after surgery with no PoTF changes.

**Figure 2:**
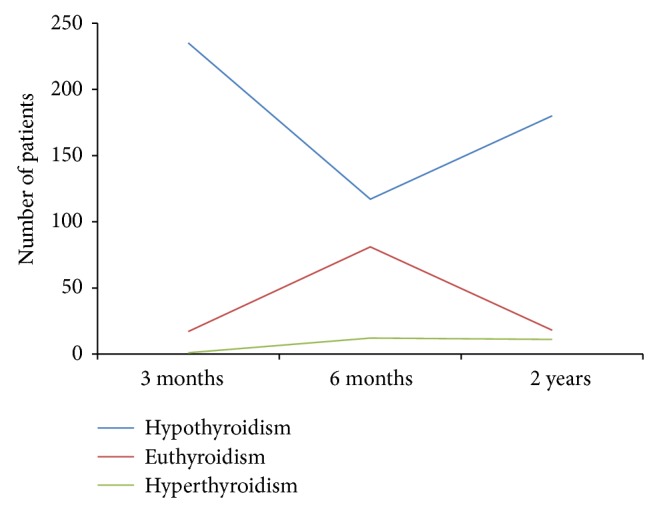
Pattern of postoperative thyroid function changes over time after subtotal thyroidectomy in Graves' disease.

Two hundred and nine ST patients came to the out-patient department for their scheduled follow-up for more than 2 years after surgery. In most of the patients with either hypothyroidism or hyperthyroidism, the change in PoTF over time was detected within the first 6 months of follow-up. After ST, hyperthyroidism developed gradually over time, while hypothyroidism, in most cases, was diagnosed within a year after surgery (Figure 2). In the ST group, 15 patients presented with euthyroidism at 3 months and, thereafter, nine of them evolved to clinical hypothyroidism, and one evolved to clinical hyperthyroidism. One hundred and ninety-three patients presented with hypothyroidism at 3 months, 13 of whom went on to develop euthyroidism and nine went on to develop clinical hyperthyroidism. Hyperthyroidism was present in one patient in whom no change in PoTF was observed during the 2 years of follow-up. No early subclinical hypothyroidism, early clinical hyperthyroidism, or late subclinical hyperthyroidism was detected during the study period (Table 3). Among the 209 ST patients who complied with the scheduled follow-up protocol, 11 (5.2%) developed clinical hyperthyroidism over time that qualified as GD recurrence requiring additional therapy such as antithyroid drug therapy (*n* = 7) or RAI therapy (*n* = 4). By contrast, no recurrence was observed in the TT group. No significant difference in surgical complication rate was observed between the ST and TT groups (Table 4).

## 4. Discussion

The most acknowledged benefit of ST in GD is maintenance of PoTF in euthyroidism after surgery. Palit et al. [8] meta-analyzed 35 studies of ST in GD and reported that an average of 59.7% of the patients presented with euthyroidism after surgery; however, the rate of euthyroidism or postoperative thyroid dysfunction varies greatly, with hypothyroidism observed in 4.0–83.0% of the cases and recurrence in 1.0–14.6% of the cases [12, 14, 16–19].

Thus, there are many reasons for the wide range of postoperative thyroid dysfunction after ST. First, the definition of dysfunction can differ based on PoTF range criteria. When hypothyroidism or hyperthyroidism is defined as abnormal TFT without being correlated with patient symptoms, the rate of dysfunction may increase. Secondly, the amount of remnant thyroid may differ between surgeons, and procedures for measuring the weight of remnant thyroid may vary between institutions; as a result, the PoTF may change even though the same amount of remnant thyroid weight is recorded. Thirdly, the iodine intake may differ between populations and could affect the PoTF. The rate of postoperative hypothyroidism is lower in regions with high intake of iodine [20]. Koreans take in high levels of iodine since the peninsula is surrounded by the ocean on three sides. Nonetheless, a high rate of hypothyroidism after ST was observed in this study.

Recent studies published in the 2000s reported increased rate of dysfunction after ST in GD more frequently than did earlier studies [12, 14]. Lal et al. [14] reported a high postoperative hypothyroidism rate, up to 83.0%, the reason for which could be the strict application of hypothyroidism criteria or an insufficient amount of remnant thyroid. Another important reason for thyroid dysfunction after surgery for GD is the duration of follow-up and level of compliance and how accurately those parameters were measured. The technical accuracy of TFT has increased over time, and results are now uniformly applied worldwide, which may have increased the detection of thyroid dysfunction after surgery [21]. Thyroid function changes over time, as long observation periods have shown [22].

In this study, most of the hypothyroidism occurred within several months after ST, and incidence decreased thereafter; by contrast, recurrence gradually increased over time although recurrence rates were low. As in the study by Noh et al. [18], most ST patients presented hypothyroidism in early postoperative TFT, and a gradual increase in the recurrence rate was observed upon long-term follow-up. Only few patients maintained euthyroidism after ST. Also, such results might have been derived from the condition that the patients after TT have received an adequate amount of thyroid hormone medication based on the generalized dosage protocol according to their body weight, and instead in ST, patients would have required adjustment duration to reach the adequate thyroid hormone levels. These results may suggest that TT is a better surgical option than ST in GD since no PoTF fluctuation or recurrence is observed after TT.

One may argue that TT presents a potentially high risk of surgical complications. Studies comparing surgical complications between ST and TT in GD showed a higher rate of transient complications but no significant differences in permanent complications. Previous reports showed that the surgical complication rate was higher after TT than after ST [12–15]; however, in this study, the overall rate of complication was higher in ST than in TT, mostly of transient hypoparathyroidism, although no statistical significance was reached. This might be explained by the overall resected thyroid weight difference. The ST group median thyroid weight was measured larger than the TT group, maybe because one of the reasons for TT was suspicion of malignancy rather than the symptomatic large goiter. Fortunately, no recurrent laryngeal nerve injury was observed during the study period, likely a consequence of the fact that a single experienced surgeon performed the surgery even though routine laryngoscopy was not used. These findings, including that of a lower complication rate in TT than ST, could explain why preference for TT over ST has increased over time. Interestingly, even though severe ophthalmopathy was a TT indicator in our institution, the rate of ophthalmopathy was higher in ST group than TT group (29.1 versus 17.7%). Fortunately, none of the patients presented detectable progression of ophthalmopathy after the surgery. The limitation of this study may be that the number of patients who underwent ST was larger than the number who underwent TT, affecting the comparison of complication risk and the suggestion that complication risk explains the trend toward greater selection of TT over ST over time.

## 5. Conclusion

In ST performed for GD, the rate of hypothyroidism in early PoTF was very high with only few patients presenting with euthyroidism. Indeed, recent studies reported a higher rate of postoperative thyroid dysfunction in ST than in TT, with increased dysfunction over time. In addition, no significant difference in surgical complication rates was observed between ST and TT. Therefore, to reduce potential long-term dysfunction and GD recurrence after surgery, TT may be more beneficial than ST in GD requiring surgery in experienced endocrine surgeon.

## Figures and Tables

**Table 1:** Clinical characteristics of Graves' disease patients who underwent surgery during the study period.

Clinical characteristics	All		Subtotal thyroidectomy		Total thyroidectomy	
	(*n* = 350)		(*n* = 254)		(*n* = 96)	
	*n*	%	*n*	%	*n*	%
Gender						
Female	270	77.0%	185	72.8%	85	88.5%
Male	80	22.9%	69	27.2%	11	11.5%
Age at surgery (year): median, IQR^†^	30	(24, 39)	28	(23, 34)	39	(31.3, 50)
Resected thyroid weight (gram): median, range	70.6	(12.0–361.0)	84.9	(13.8–361.0)	32.5	(12.0–160.0)
Follow-up duration after surgery (month): median, IQR^†^	61	(24, 103.3)	57	(23, 107)	63.5	(28.3, 91.8)
Ophthalmopathy						
No	259	74.0%	180	70.9%	79	82.3%
Yes	91	26.0%	74	29.1%	17	17.7%
Reasons for surgery						
Resistance to ATD^‡^	195	55.7%	179	70.5%	16	16.7%
Symptomatic large goiter	24	6.9%	22	8.7%	2	2.1%
Suspicious undetermined mass	21	6.0%	12	4.7%	9	9.4%
Suspicion of malignancy	67	19.1%	0		67	69.8%
Patient's desire	33	9.4%	31	12.2%	2	2.1%
Others (not described)	10	2.90%	10	3.9%	0	
Incidence of thyroid carcinoma presented^∗^	79	22.6%	5	2.0%	74	77.1%
Papillary thyroid carcinoma size (cm): median, range	0.83	(0.2–5.2)	0.38	(0.3–0.5)	0.88	(0.2–5.2)

^†^IQR: interquartile range; ^‡^ATD: antithyroid hormone drug.

^∗^All the thyroid carcinomas presented in Graves' disease were papillary thyroid carcinomas.

**Table 2:** Postoperative thyroid function after subtotal thyroidectomy at 3 months and thyroid function distribution by amount of remnant thyroid.

Postoperative thyroid function			Remnant thyroid weight						*p*
	Subtotal thyroidectomy (*n* = 254)		<4.0 g		≥4.0 g and <6.0 g		≥6.0 g		
			(*n* = 73)		(*n* = 153)		(*n* = 28)		
	*n*	%	*n*	%	*n*	%	*n*	%	
Hypothyroidism	235	92.5%	69	95.8%	141	92.2%	25	89.3%	0.671
Euthyroidism	17	6.7%	3	4.2%	11	7.2%	3	10.7%	
Hyperthyroidism	1	0.4%	0		1	0.7%	0		

**Table 3:** Change in postoperative thyroid function in 209 Graves' disease patients after subtotal thyroidectomy 3 months and 2 years after surgery.

			Follow-up: 2 years			
Postoperative thyroid function			Hypothyroidism		Euthyroidism (*n* = 18)	Hyperthyroidism Clinical (*n* = 11)
			Subclinical (*n* = 9)	Clinical (*n* = 171)		
Follow-up: 3 months	Hypothyroidism	Clinical (*n* = 193)	9	162	13	9
	Euthyroidism (*n* = 15)		0	9	5	1
	Hyperthyroidism	Subclinical (*n* = 1)	0	0	0	1

**Table 4:** Comparison of surgical complication rates between subtotal thyroidectomy and total thyroidectomy in Graves' disease.

Complications	Subtotal thyroidectomy		Total thyroidectomy		*p*
	(*n* = 254)		(*n* = 96)		
	*n*	%	*n*	%	
Complications (total)					0.089
No	222	87.4%	90	93.8%	
Yes	32	12.6%	6	6.3%	
Types of complication					
Postoperative bleeding requiring surgical exploration	6	2.4%	2	2.1%	
Transient hypoparathyroidism	24	9.4%	4	4.2%	
Permanent hypoparathyroidism	1	0.4%	0		
Recurrent laryngeal nerve injury	0		0		
Wound seroma	1	0.4%	0		
